# Outcome of robot-assisted pancreaticoduodenectomy during initial learning curve versus laparotomy

**DOI:** 10.1038/s41598-020-66722-2

**Published:** 2020-06-15

**Authors:** Jiangjiao Zhou, Li Xiong, Xiongying Miao, Juan Liu, Heng Zou, Yu Wen

**Affiliations:** Department of General Surgery, The Second Xiangya Hospital, Central South University, No.139 Middle Renmin Road, Changsha, Hunan 410011 P.R. China

**Keywords:** Pancreatic cancer, Surgical oncology

## Abstract

To analyze the initial learning curve (LC) for robot-assisted pancreaticoduodenectomy (RAPD) and compare RAPD during the initial LC with open pancreaticoduodenectomy (OPD) in terms of outcome. This study is a retrospective review of patients who consecutively underwent RAPD and OPD between October 2015 and January 2020 in our hospital. 41 consecutive RAPD cases and 53 consecutive open cases were enrolled for review. Compared with OPD, RAPD required a significantly longer operative time (401.1 ± 127.5 vs. 230.8 ± 44.5 min, *P* < 0.001) and higher cost (194621 ± 78342 vs. 121874 ± 39973 CNY, *P* < 0.001). Moreover, compared with the OPD group, the RAPD group revealed a significantly smaller mean number of lymph nodes harvested in malignant cases (15.6 ± 5.9 vs 18.9 ± 7.3, *P* = 0.025). No statistically significant differences were observed between the two groups in terms of incidence of Clavien–Dindo grade III–V morbidities and 90-day mortality and readmission (P>0.05). In the CUSUM graph, one peak point was observed at the 8th case, after which the operation time began to decrease. LC for RAPD may be less than 30 cases, and RAPD is safe and feasible during the initial LC.

## Introduction

Although pancreatectomy was first performed 80 years ago, it remains a challenging abdominal surgery with relatively high morbidity and mortality^1^. Laparoscopic pancreaticoduodenectomy (LPD) was first reported in 1994 by Gagner^2^; today, this procedure could be performed as safely as open pancreaticoduodenectomy (OPD) by skilled surgeons^3,4^. However, the long learning curve (LC) for LPD continues to challenge many surgeons. In general, LPD presents intrinsic disadvantages compared with conventional laparotomy, including instrument motion, two-dimensional imaging, poor surgeon ergonomics, and a long LC. In 2000, the Da Vinci system was approved by the Food and Drug Administration. In 2003, Giulianotti *et al*. published a case series verifying the feasibility of robotic pancreatectomy; the series included eight robot-assisted pancreaticoduodenectomy (RAPD) and five robot-assisted distal pancreatectomy (RADP) cases^5^. Robotic surgery is an advanced minimally invasive surgical technique that has several benefits in pancreaticoduodenectomy, such as enhanced three-dimensional vision, application of EndoWrist instruments (which have a great range of motion), and a short LC. However, the safety of RAPD during the initial LC and the possible shortening of the LC remain unclear. Therefore, this article addresses the LC of a single surgical team in our hospital.

## Methods

### Patient Selection

This study is a retrospective research on pancreaticoduodenectomy conducted by a single surgical team. Our team had performed over 200 OPD before October 2015 at the Department of Pancreatic Surgery (The Second Xiangya Hospital, Central South University, Changsha, China). In October 2015, our hospital installed Da Vinci Si Robot Surgical System. All 41 RAPD and 53 OPD cases performed by our team between January 2016 and January 2020 were included in this study. All cases were confirmed resectability on the basis of preoperative radiology, we followed the criteria defining resectability status of *NCCN Guidelines for Pancreatic Adenocarcinoma*, only cases that meet the resectable criteria was administrated for OPD or RAPD. Otherwise, patients were excluded for surgery. The exclusion criteria for RAPD were as follows: (1) maximum circumferential diameter of tumor greater than 5 cm, (2) any suspicious invasion of the superior mesenteric artery/vein, (3) body mass index ≥28 kg/m^2^, and (4) patients with severe cardiorespiratory comorbidities that cannot tolerate RAPD. Informed consent was waived by the Ethics Committee of The Second Xiangya Hospital. All methods in this study were performed in accordance with the declaration of Helsinki.

### Perioperative cure

All patients underwent preoperative routine examination (routine blood tests, chest X-ray, electrocardiogram), tumor markers (cancer antigen 19-9, carcinoembryonic antigen, and α-fetoprotein), abdominal computed tomography, or magnetic resonance imaging. Percutaneous transhepatic cholangio drainage was performed when total bilirubin exceeded 250 umol/L. On the second postoperative day, the nasogastric tube was removed if drainage was less than 200 ml/d. Prophylactic antibiotic treatment was used for 3 days, and prophylactic somatostatin was given for at least 7 days. Self-administered analgesia, including 100 μg of sufentanil, 10 mg of butorphanol tartrate, and 16 mg of ondansetron hydrochloride, was given to 3 days after surgery. The amylase level of the peripancreatic drainage fluid was measured for the first time on the third day after surgery and then recorded every 2 days thereafter.

### Surgical technique

**Figure 1:**
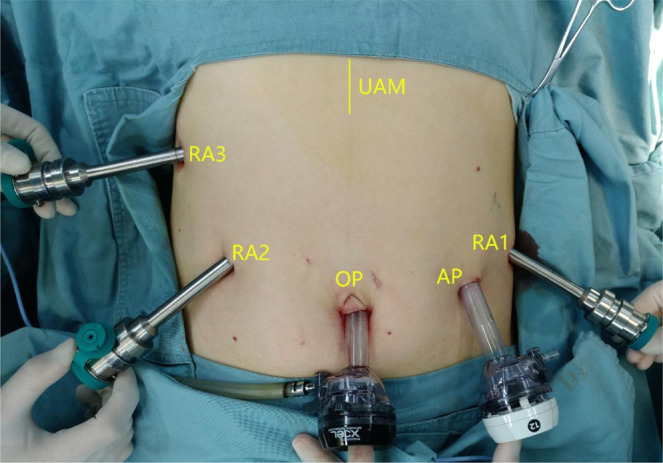
Placement of the 5 ports. RA1: 8-mm trocar along the left anterior axillary line; RA2: 8-mm trocar along the right midclavear line; RA3: 8-mm trocar along the right anterior axillary line; The optic port (OP):12-mm trocar under umbilicus; The assistant port (AP):12-mm trocar along the left midclavear line. UAM: Upper abdomen midline incision.

All RAPD and OPD cases were performed by the same surgical team. The operator was Yu Wen, and the first assistant was Jiangjiao Zhou or Heng Zou. Before we started RAPD in January 2016, Wen Yu had already went to University of Pittsburgh Medical Center in February 2012 and learned robotic pancreatic surgery for one year. Also we had finished more than 20 other simple robotic surgery such as cholecystectomy and distal pancreatectomy. These experiences helped us a lot at the beginning. For RAPD, the patients were placed on their back with the head and right side raised by 30°. The location of the trocar is shown in Fig. 1.

Extensive exploration of the abdominal cavity and pelvis was performed before the start of the operation, and any nodule considering metastasis was biopsied. The gastrocolic omentum was opened with an ultrasonic scalpel (Ethicon), and the hepatic flexure and transverse colon were pushed downward to reveal the pancreatic head and the duodenum. The duodenum was then mobilized using the Kocher maneuver until the aorta was exposed, and the stomach was transected 6–10 cm along the left side of the pylorus with an endo linear stapler. The gastric duodenal artery and the right gastric artery were clipped at the root, and the lymph nodes around the hepatic artery and hepatic hilum were removed. Next, cholecystectomy was performed, and the common hepatic duct was exposed and incised. The lower edge of the pancreas was separated, the superior mesenteric and portal veins were identified, and the posterior pancreatic tunnel was established. The pancreas was disconnected by the ultrasonic scalpel, and the main pancreatic duct was cut using scissors. Afterward, the jejunum was pulled to the right side of the superior mesenteric vein. The distal jejunum was divided at a distance of 10–15 cm to the duodenojejunal flexure using an endo linear cutting stapler (Ethicon). The head of the pancreas and duodenum were removed by the ultrasonic scalpel (Ethicon). The pancreatic uncinate process was dissociated along the right aspect of the superior mesenteric vein until the specimen was completely removed.

Digestive tract reconstruction was performed using Child’s method. The order of anastomosis was as follows: pancreaticojejunostomy → hepaticojejunostomy → gastrojejunostomy. Gastrointestinal anastomosis reconstruction was performed behind the transverse colon. Pancreaticojejunostomy was performed from the pancreatic duct to the jejunal mucosa. A pancreatic duct catheter was placed in the pancreatic duct to drain the pancreatic juice to the distal jejunum of the pancreatic intestine. The biliary anastomosis was continuously sutured by an absorbable barb wire without T tube placement. Gastrointestinal anastomosis was performed by a linear cutting occluder to achieve side-to-side anastomosis between the posterior wall of the stomach and jejunum. After the operation, drainage tubes were placed under the Winslow hole and the pancreatic intestine anastomosis, which were individually taken out from both sides of the abdominal wall. Specimens were obtained through a 3 cm incision in the upper abdomen midline incision.

### Date collection

All perioperative information was collected retrospectively for analysis, including the following variables:

1. Baseline characteristics: age, gender, initial symptoms, comorbidities, and American Society of Anesthesiology (ASA) score;
2. Surgical information: operative time, transfusion rate, and conversion rate; and
3. Postoperative data: total medical expenses, postoperative hospital stay, final pathologic results, short-term complications, and 90-day mortality and readmission.

For tumor cases, pathological dates were recorded by tumor-node-metastasis staging, as recommended by the AJCC Cancer Staging Manual (8th Edition). Postoperative complications were classified by the Clavien–Dindo classification of surgical complications^6^. Furthermore, morbidities were defined and graded following the criteria drafted by the International Study Group of Pancreatic Surgery, including delayed gastric emptying, pancreatic fistula, and hemorrhage^7,8^.

### Statistical analysis

The statistics software SPSS (version 25.0, SPSS Inc., Chicago, IL, USA) was used for data analyses. Continuous variables were expressed as mean ± standard deviation (SD), and categorical variables were presented as numbers and percentages. The mean values of continuous variables were compared by either the two-tailed Student’s *t*-test or the nonparametric Wilcoxon rank-sum test. Categorical variables were compared by using Pearson’s χ^2^ test or Fisher’s exact test contingency tables, and Student’s *t* test was used to compare data between the groups. Statistical significance was defined as *P* < 0.05.

#### Cumulative sum (CUSUM) method

The LC of RAPD was calculated using CUSUM. CUSUM_OT_, which refers to the difference between the operation time of the first patient and the mean OT of all patients, is calculated as CUSUM_OT_ = Σ^n^_i_ = 1 (x_i_ − μ), where μ is the median overall operation time and xi is an individual operation’s time. Notable change points were identified at the point showing the largest peak in the CUSUM curve.

### Ethics approval and consent to participate

The study was approved by the Ethics Committee of The Second Xiangya Hospital.

## Results

### Details of patients and pathology

In total, 41 cases and 53 cases were scheduled for RAPD and OPD, respectively, between January 2016 and January 2020. These procedures were performed by one experienced pancreatobiliary surgical team. All 41 RAPD cases were considered the initial LC.

The baseline characteristics of the patients are summarized in Table 1. The mean age of the RAPD group (18 male and 23 female) was 58.2 ± 10.5 years. In addition, 20 (46.7%) and 21 (61%) patients were classified as ASA II and III, respectively. In the OPD group (31 male and 22 female), the mean age was 58.1 ± 9.9 years. In total, 1 (1.9%), 26 (49.1%), and 26 (49.1%) patients were classified as ASA I, II, and III, respectively. Pathologic diagnoses included malignant lesions in 80.5% (33/41) of the RAPD cases, including pancreatic cancer (n = 14), duodenum carcinoma (n = 11), ampullary carcinoma(n = 2), the terminal bile duct carcinoma (n = 2), neuroendocrine carcinoma of duodenum(n = 1) and pancreatic cystadenocarcinoma (n = 3), and in 88.7% (47/53) of the OPD cases, including pancreatic cancer (n = 25), duodenum carcinoma (n = 12), the terminal bile duct carcinoma (n = 5), neuroendocrine carcinoma of duodenum(n = 1) and pancreatic cystadenocarcinoma (n = 4). Benign lesions for RAPD group were heterotopic pancreas of duodenum (n = 1), pancreatic cystadenoma (n = 2), neuroendocrine tumor of duodenum (n = 2), duodenal adenoma (n = 3); for OPD group, it were pancreatic cystadenoma (n = 4), chronic pancreas inflammatory mass (n = 1). Jaundice, followed by epigastric pain, was the most common chief complaint of the RAPD and OPD groups. No statistically significant differences between the two groups were observed in terms of age, gender, comorbidity, and ASA score (P >0.05).

**Table 1:** Demographic and comorbidity characteristics of all patients.

Characteristic	RAPD	OPD	P value
Age, yr (range)	58.2 ± 10.5 (34–76)	58.1 ± 9.9 (25–77)	0.957
Male/female	18/23	31/22	0.16
Malignant/benign	33/8	47/6	0.269
Comorbidities			0.9
Diabetes	3	4	
Hypertension	4	4	
Cardiovascular diseases	0	1	
Cerebrovascular disease	1	0	
Pulmonary disease	0	1	
Chronic pancreatitis	0	1	
Cirrhosis	1	0	
ASA score			0.710
ASA 1	0	1	
ASA 2	20	26	
ASA 3	21	26	

OPD: Open pancreaticoduodenectomy; RAPD: Robot-assisted pancreaticoduodenectomy;

ASA: American Society of Anesthesiologists.

### Intraoperative and postoperative outcomes

The mean operative time in the RAPD group was much longer than that in the OPD group (*P* < 0.001) (Table 2). No statistical difference between the two groups was identified in terms of perioperative transfusion. The mean number of lymph nodes harvested in the RAPD group was fewer than that in the OPD group (*P* = 0.025). All patients underwent R0 resection. The incidence of pancreatic fistula between the two groups was not statistically different. According to the Clavien–Dindo classification, the rates of grades I–V in the RAPD group were lower (24/41, 58.5%) than those in the OPD group (31/53, 58.5%) (*P* = 0.38), and no statistically significant difference between the two groups was found in terms of rates of grades III–V incidence (RAPD: 12/41, 29.3%; OPD: 14/53, 26.4%; *P* = 0.759). Moreover, no difference between the two groups was observed in terms of 90-day mortality and readmission. Patients in the RAPD group (23.6 ± 19.1) had longer postoperative hospital stays compared with those in the OPD group (20.3 ± 13.4), but the difference was not statistically significant (*P* = 0.328). As expected, the total cost of pancreaticoduodenectomy in the RAPD group was much higher than that in the OPD group (194621 ± 78342 versus 121874 ± 39973 CNY, *P* <0.001) (Table 2).

**Table 2:** Intraoperative and postoperative information among the learning curve phases.

Characteristic	RAPD	OPD	P value
Operative time (min)	401.1 ± 127.5	230.8 ± 44.5	<0.001
Perioperative transfusion needed, n	9(21.8%)	9(17%)	0.544
No. of lymph nodes harvested	15.6 ± 5.9	18.9 ± 7.3	0.025
Length of hospital stay (d)	23.6 ± 19.1	20.3 ± 13.4	0.328
Clavin-Dindo, n	24(58.5%)	31(58.5%)	0.38
I	3(7.3%)	11(20.8%)	
II	11(26.8%)	8(15.1%)	
IIIA	4(9.8%)	7(13.2%)	
IIIB	2(4.9%)	3(5.7%)	
IV	2(4.9%)	0	
V	2(4.9%)	2(3.8%)	
Clavin-Dindo ≥III, n	12(29.3%)	14(26.4%)	0.759
Pancreatic fistula	20(48.8%)	18(34%)	0.437
A	11(26.8%)	12(22.6%)	0.640
B	7(17.1%)	5(9.4%)	0.271
C	2(4.9%)	1(1.9%)	0.821
Mortality (90-day)	2(4.9%)	2(3.8%)	>0.9
Readmission (90-day)	1(2.4%)	2(3.8%)	>0.9
Fee (CNY)	194621 ± 78342	121874 ± 39973	<0.001

CNY: Chinese Yuan; OPD: Open pancreaticoduodenectomy; RAPD: Robot-assisted pancreaticoduodenectomy.

### LC analysis

**Figure 2:**
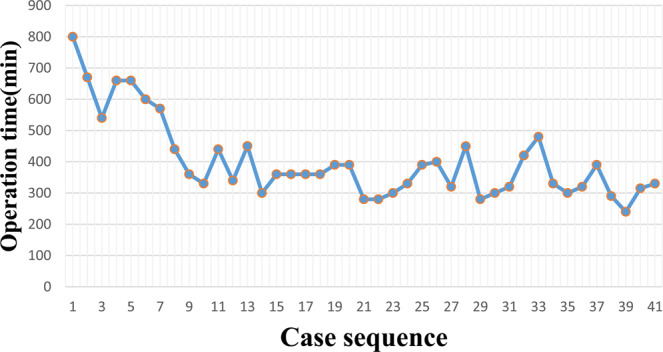
Graph of operative times plotted for each of the 41 consecutive patients.

**Figure 3:**
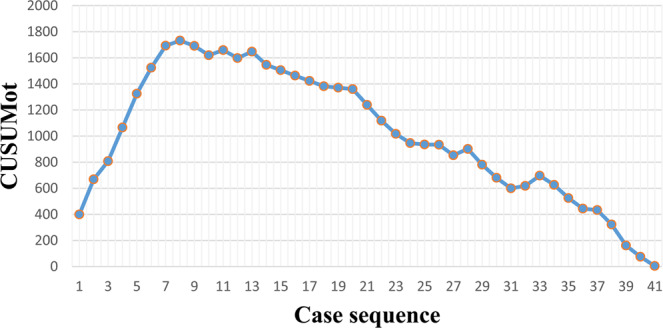
Cumulative sum graph for operative time.

The operation time was calculated for each case (Fig. 2). A significantly negative correlation was identified between the number of RAPD cases experienced and operation time. The operation time was rapidly reduced from the 1st to the 10th RAPD cases, fluctuated slightly between the 10th and 14th cases, and became relatively stable from the 15th to the 41st cases. The LC was assessed by the CUSUM method. In the CUSUM_OT_ graph (Fig. 3), one peak point was observed at the 8th case, after which the operation time began to decrease.

## Discussion

Minimally invasive pancreaticoduodenectomy (MPD) includes LPD and RAPD. LPD was first described by Gagner and Pomp in 1994^2^, while RAPD was first performed by Giulianotti *et al*. in 2001^5^. LPD is a safe and effective procedure in specialized high-volume medical centers^4,9,10^. However, traditional laparoscopy systems are associated with intrinsic disadvantages, including two-dimensional visualization, poor surgeon ergonomics, and a restricted range of movement (up to only four degrees of freedom) inside the abdominal cavity due to the straight bodies of laparoscopic instruments. Some components of the procedure, such as pancreatic enteric reconstruction, are technically demanding because of these limitations. Robotic systems provide surgeons with superior three-dimensional visualization and instrumentation that mimics the latter’s hands; these instruments have an articulating wrist, can achieve seven degrees of freedom, and provide tremor filtration and stable retraction. Given the above advantages of robotic systems, surgeons can control surgical instruments flexibly, accurately, and with a wide range of motion, which is critical for operations requiring complex resection and reconstruction. Such operations include pancreaticoduodenectomy, which entails considerable suturing and knotting due to the need for pancreaticojejunostomy, hepaticojejunostomy, and gastrojejunostomy. Therefore, RAPD surgery is more advantageous than LPD surgery. Recent reports have shown that RAPD is safer and more efficient than LPD among properly selected patients^11,12^. Therefore, RAPD is technically a feasible alternative to the laparoscopic procedure. Further studies may be needed to evaluate the cost-effectiveness of RAPD^13^.

To the best of our knowledge, four reports have been published on the LC for RAPD. Napoli *et al*. reported decreased operation times and postoperative gastric emptying delay rates after an 33 initial RAPD procedures^14^. Liu *et al*. divided the LC of RAPD into two parts; the operation time first decreased after 20 cases of RAPD and then decreased again after 20 additional cases (*P* < 0.01)^15^. Therefore, the authors believe that the LC is completed after 40 RAPD procedures^15^. Boone *et al*. and Chen *et al*. described their LC in RAPD^16,17^. Boone *et al*. completed 200 cases of RAPD and found that the operation time was significantly reduced after the 80th operation^16^. Chen *et al*. designed a non-randomized prospective case-control study comparing 120 OPD with 60 RAPD cases and found that the average postoperative time of the last 20 RAPD patients was significantly shorter than that of the first 40 cases^17^. As shown by the results of these four studies, the LC is completed within at least 33 RAPD cases. The present research included 41 RAPD procedure, peak points in the CUSUM_OT_ graph (Fig. 3) were observed at the 8th case. In our opinion, if we can learn from the experiences of other surgical teams well, we can shorten the LC.

According to previous reports, the LC of OPD corresponds to 50–60 operations^18–20^. Speicher *et al*. reported that OT and blood loss could be reduced after an initial 50 cases of LPD^21^. However, RAPD should theoretically have a shorter LC than LPD due to the advantages of the robotic system^22^. As shown by the results of the present and the four previous studies, RAPD can be safely performed at a specialized pancreatic surgery center, and each surgeon will require approximately 30–40 cases to complete the LC. The LC for RAPD is shorter than that of either OPD^18–20^ or LPD^21^. Of course, surgeons should be rich experienced in OPD before carrying out the first RAPD.

We compared the outcomes of RAPD during our initial LC with OPD groups. No statistical differences between the two groups were found in terms of the need for perioperative transfusion, PF rate, length of stay, rate of Clavien–Dindo grade ≥ III morbidity, and rate of mortality (På 0.05). All of the patients underwent R0 resection, and no case conversion to open surgery occurred in the RAPD group. The mean operative time was longer, the mean number of lymph nodes harvested was fewer, and total cost was much higher in the RAPD group than in the OPD group. According to the LC, we divided 1–8 cases into the first phase and 9–41 cases into the second phase. We made a subgroup analysis for the number of lymph nodes harvested between the first stage phase [15.9 ± 6.2] and the second [14.0 ± 3.8]. There was no significant difference (P = 0.485). Therefore, while RAPD is safe during the initial LC, additional patients and resources may be necessary due to its long operation time and high cost.

We also compared the outcome of RAPD during the initial LC with the results from the four previous reports^14–17^ (Table 3). The mean operation time ranged from 401 min to 581 min. Boone *et al*.^16^ reported 9 days as an unusually short length of hospital stay. However, this finding may be due to differences in medical systems across countries. Unlike in the United States^16^, patients in China who do not recover completely cannot easily be arranged for discharge. In two cited reports from China^15,17^, the mean length of hospital stay exceeded 20 days. Our number of harvested lymph nodes was relatively small. Interestingly, the numbers of harvested lymph nodes reported by the cited studies from China^15,17^ were 6.42 and 13.6, respectively, similar to our results and significantly fewer than the 36.8 and 22 reported by Italian^14^ and American^16^ scholars, respectively. We suggest that, on the one hand, Chinese surgeons should give much more attention to lymph node dissection. On the other hand, pathologists need to be more patient with specimens of pancreatoduodenectomy.

**Table 3:** Comparison of RAPD learning curve with previous studies.

Research	Zhou	Zhang	Napoli	Boone	Chen
Cases before complete LC	41	40	33	80	40
Operative time, min	401.1	418	564.7	581	445
Conversion to open	4.9%	10%	0.0%	11.2%	1.7%
Length of hospital stay	23.6	22	22.6	9	20
Post-operative complications	58.5%	65.0%	78.8%	67.5%	35.0%
Clavien-Dindo ≧III	29.3%	30.0%	12.1%	26.0%	11.7%
Postoperative mortality	4.9%	7.5%	3.0%	3.3%	1.7%
Margin negative resection	100%	100%	100%	92.00%	97.80%
Lymph nodes harvested	15.6	6.42	36.8	22	13.6

RA1: 8-mm trocar along the left anterior axillary line;

RA2: 8-mm trocar along the right midclavear line;

RA3: 8-mm trocar along the right anterior axillary line;

The optic port (OP):12-mm trocar under umbilicus;

The assistant port (AP):12-mm trocar along the left midclavear line.

UAM: Upper abdomen midline incision.

Our analysis has several limitations. First, the followup should be extended, progression-free survival and overall survival need to be verified. Second, surgeons with experience in LPD may have a short LC for RAPD. By contrast, our team has no experience in LPD. Third, this research is a retrospective study. Thus, the integrity and homogeneity of research data cannot be guaranteed.

## Conclusions

In conclusion, RAPD can be safely performed in well-selected patients during the initial LC by pancreatobiliary surgeon teams with extensive experience in OPD surgery. LC for RAPD may be less than 30 cases.

## Data Availability

The datasets used and analysed during the current study are available from the corresponding author on reasonable request.
